# Pixel-Wise Sky-Obstacle Segmentation in Fisheye Imagery Using Deep Learning and Gradient Boosting

**DOI:** 10.3390/jimaging11120446

**Published:** 2025-12-12

**Authors:** Némo Bouillon, Vincent Boitier

**Affiliations:** LAAS-CNRS, Université de Toulouse, CNRS, 31400 Toulouse, France; vboitier@laas.fr

**Keywords:** deep learning, convolutional neural networks (CNN), fisheye imagery, multiscale segmentation, data augmentation, sky segmentation, sky imaging, gradient boosting

## Abstract

Accurate sky–obstacle segmentation in hemispherical fisheye imagery is essential for solar irradiance forecasting, photovoltaic system design, and environmental monitoring. However, existing methods often rely on expensive all-sky imagers and region-specific training data, produce coarse sky–obstacle boundaries, and ignore the optical properties of fisheye lenses. We propose a low-cost segmentation framework designed for fisheye imagery that combines synthetic data generation, lens-aware augmentation, and a hybrid deep-learning pipeline. Synthetic fisheye training images are created from publicly available street-view panoramas to cover diverse environments without dedicated hardware, and lens-aware augmentations model fisheye projection and photometric effects to improve robustness across devices. On this dataset, we train a convolutional neural network (CNN) and refine its output with gradient-boosted decision trees (GBDT) to sharpen sky–obstacle boundaries. The method is evaluated on real fisheye images captured with smartphones and low-cost clip-on lenses across multiple sites, achieving an Intersection over Union (IoU) of 96.63% and an F1 score of 98.29%, along with high boundary accuracy. An additional evaluation on an external panoramic baseline dataset confirms strong cross-dataset generalization. Together, these results show that the proposed framework enables accurate, low-cost, and widely deployable hemispherical sky segmentation for practical solar and environmental imaging applications.

## 1. Introduction

### 1.1. Context and Motivation

Long-term resource assessment is a critical step in photovoltaic system design, since developers need reliable estimates of how much solar energy will be available at a site, often in complex environments where surrounding obstacles such as trees, poles, or nearby buildings create shading. Traditional approaches often rely on digital surface models [1], but their ability to represent these local features is limited by data resolution and coverage.

Sky imaging provides an interesting alternative by using fisheye cameras to capture the entire sky hemisphere in a single frame. These images record both the visible sky and the surrounding obstacles, providing the basis to study how obstructions affect solar access. Fisheye lenses are available in both high-end, professional models and inexpensive consumer versions; the latter include low-cost clip-on smartphone adapters that are commercially available for less than 30 USD, which make fisheye imagery easy to acquire in practice.

These fisheye images not only capture the visible sky but also provide the information needed for quantitative solar assessment. In solar engineering, global irradiance is commonly described as the sum of direct beam, diffuse sky, and ground-reflected components [2]. Accurate sky–obstacle segmentation from fisheye imagery enables the estimation of direct and diffuse irradiance components by computing shading factors that describe the fraction of the sky dome obstructed by surrounding objects. Specifically, one shading factor corresponds to the direct component, obtained by tracing the sun’s path and identifying obstructions, and another corresponds to the diffuse component, derived from the proportion of the sky covered by obstacles. These coefficients quantify the irradiance loss due to shading and thus allow estimation of effective irradiance from fisheye images [3].

To support such analyses, a fisheye-specific segmentation framework is introduced that is low cost, accurate, and generalizable across diverse environments. The framework is further integrated into a shading estimation tool designed to remain simple and practical for end users.

Before reviewing prior work, two specific issues are highlighted as major obstacles for fisheye-based sky segmentation: the unique distortions introduced by fisheye optics, and the inadequacy of existing public datasets.

### 1.2. Challenges of Fisheye Imagery

Compared to conventional pinhole or rectilinear lenses, fisheye optics use a highly non-linear projection to map an extremely wide field of view onto the image plane. This mapping introduces strong radial distortion that increases toward the image periphery. Rather than preserving straight lines and geometric proportions, fisheye lenses deliberately trade spatial fidelity for maximum scene coverage.

The fisheye imaging process is typically modeled as a two-step projection onto a virtual unit sphere. First, 3D scene points are projected linearly onto the unit sphere. Then, the spherical points are mapped onto the image plane through a nonlinear projection function. Four projection models are commonly used to describe this transformation: the equidistant, equisolid-angle, orthographic, and stereographic projections [4].

These structural properties pose considerable challenges for conventional image processing methods. First, the distortion alters object shapes, making it difficult to apply models pretrained on undistorted datasets. Second, the image borders often exhibit degraded visual quality due to optical effects such as astigmatism, blur, and chromatic aberration [5], all of which can impair the predictions of segmentation models not specifically adapted to these distortions.

### 1.3. Limitations of Public Datasets for Fisheye Sky Segmentation

Despite the growing availability of public datasets for semantic segmentation, existing resources are largely inadequate for our target task. Most publicly available datasets such as Cityscapes [6], ADE20K [7], and ApolloScape [8] are designed for autonomous driving or general scene parsing and typically employ rectilinear camera systems. Consequently, fisheye imagery in these datasets is either absent or captured in a manner that does not represent a true hemispherical view of the sky. Moreover, these datasets often focus on object-centric or urban scene segmentation rather than sky–obstacle discrimination.

Furthermore, when sky annotations are available in these datasets, they tend to be coarse, with imprecise boundaries that are inadequate for applications requiring fine-grained pixel-level segmentation of sky–obstacle boundaries. Occlusions such as tree branches, cables, or architectural elements near the skyline are often simplified or excluded from annotation masks. Additionally, most public datasets are geographically biased toward urban environments in Europe, North America, or China, limiting their representativeness in rural, mountainous, or climatically diverse locations.

### 1.4. Contributions

These observations highlight the need for approaches that are both robust across diverse imaging conditions and capable of precise, pixel-accurate sky–obstacle delineation required for shading-factor estimation. To address these gaps, our work makes three main contributions:

1. A publicly released, globally diverse dataset comprising 182 fisheye images: 80 for CNN training and 60 for meta-model refinement, all generated from Google Street View panoramas, and 21 real smartphone images each for validation and testing. Only the corresponding annotations are publicly provided, along with a script that allows users to retrieve the original panoramas through the official Google Street View API. The real captured images used for validation and testing are released in full to ensure reproducibility and systematic benchmarking.
2. Lens-aware augmentation strategies that simulate chromatic aberration and projection variability, improving robustness to the optical distortions of real fisheye systems. They contribute an IoU increase of +0.14% and significantly enhance generalization across heterogeneous lenses and imaging conditions.
3. A hybrid segmentation framework that integrates a convolutional neural network with a gradient-boosted decision tree meta-model for pixel-level refinement. The meta-model yields consistent region-level gains (up to +0.89% IoU) and markedly larger improvements on boundary-focused metrics (up to +3.38% Boundary IoU and +5.14% Boundary F1) over the CNN-only baseline, showing that GBDT post-processing is an effective way to sharpen sky–obstacle contours.

### 1.5. Paper Organization

The remainder of this article is structured as follows. Section 2 reviews related works in sky segmentation and fisheye image analysis, providing context within existing research. Building on this background, Section 3 details the proposed fisheye sky segmentation pipeline, including dataset generation, augmentation strategies, and the hybrid CNN-GBDT framework. Section 4 presents quantitative benchmarks and segmentation metrics for comparison across models, together with qualitative visualizations on real fisheye test images. This section further examines the impact of individual contributions through ablation studies, evaluates performance on an external panoramic dataset, and analyzes runtime efficiency to assess practical applicability. Section 5 discusses the implications of the findings, highlights current limitations, and outlines possible directions for future work. Finally, Section 6 summarizes the contributions and main results of the study.

## 2. Related Work

### 2.1. Sky Imaging for Irradiance Forecasting

Recent advances in machine learning have applied sky imaging primarily to short-term irradiance forecasting (nowcasting), where cloud detection and motion estimation are used to predict solar variability over minutes to hours [9,10,11,12,13]. For example, deep networks have been especially effective for cloud analysis, covering cloud classification and sector clustering [14] and diurnal/nocturnal cloud masks via enhanced Fully Convolutional Networks (FCNs) [15], and multi-location deep learning has shown predictive value for irradiance estimation [16]. These studies highlight the potential of combining all-sky imagery with deep learning, but their focus remains on dynamic cloud modeling and short-term prediction rather than on static obstructions and long-term resource assessment.

### 2.2. Early Approaches to Sky Segmentation

Since the focus is on long-term solar resource assessment, the key challenge is to account for static obstructions across the entire sky hemisphere, as they can attenuate direct solar radiation and reduce diffuse irradiance from the sky dome. This requires accurate sky–obstacle segmentation, which has been approached in different ways over time. Early approaches to fisheye sky segmentation relied on handcrafted cues. Classical pipelines used edge or region-based descriptors, including region classification on fisheye views [17], Hellinger-kernel distances over local descriptors [18], and edge-centric methods for solar exposure prediction [19]. While computationally efficient, these methods are sensitive to illumination conditions, exposure settings, and lens artifacts. As a result, they often yield coarse skylines and struggle with thin obstacles such as branches or cables. This sensitivity is particularly critical in applications such as hemispherical canopy photography, where contrast variations strongly affect threshold-based segmentation [20,21].

### 2.3. Learning-Based Sky Segmentation and Distortion-Aware Models

The limitations of handcrafted pipelines, particularly their limited ability to generalize across diverse imaging conditions, motivated the exploration of learning-based models that can automatically extract more robust representations from data. In rectilinear imagery, learning-based sky segmentation has been addressed with supervised classifiers trained on color and texture-based features in hazy outdoor scenes [22] and for monocular obstacle avoidance, where sky/non-sky masks provide horizon and obstacle cues for navigation [23]. More recent approaches rely on convolutional models, either to adaptively select among classical sky-segmentation algorithms [24] or to predict pixel-wise sky-ground masks for navigation imagery [25], thereby reducing manual feature design and annotation effort. However, these methods all operate on rectilinear images and do not use fisheye imagery. More recently, distortion-aware architectures have emerged that explicitly address the geometry of wide-angle optics. Methods such as deformable convolutions [26,27] and radial transformer designs [28] adapt segmentation networks to fisheye projection models, marking an important step toward high-fidelity analysis of hemispherical views. However, these models are generally developed for urban driving scenes, where the sky boundary is not the primary target and segmentation accuracy is evaluated at the object level. Our framework instead focuses on precise, pixel-wise sky segmentation across globally distributed fisheye imagery, emphasizing robust skyline fidelity rather than multi-class urban semantics. While the present method does not incorporate deformation modules, these techniques are conceptually relevant to handling fisheye distortion, and we consider their integration as a potential direction for improving sky segmentation accuracy under diverse projection geometries.

### 2.4. Cross-Domain Applications of Sky Segmentation

The relevance of sky segmentation extends well beyond solar forecasting and has become essential in fields such as navigation, positioning, and remote sensing. For example, skyline-based approaches have been used for visual navigation and localization in urban canyons [29]. In navigation and Global Navigation Satellite Systems (GNSS) remote sensing of such environments, fisheye sky segmentation has been exploited in several complementary ways to mitigate signal degradation, including FCN-based sky masks in tightly coupled GNSS/Inertial Navigation System (INS)/Vision systems [30], skymask matching between sky-pointing fisheye images and 3D city models for positioning and heading estimation [31], GNSS satellite visibility analysis and LOS/Non-Line-Of-Sight (NLOS) discrimination based on fisheye sky masks [32], and patch-based GNSS satellite state characterization from fisheye imagery [33]. Recent work has also demonstrated the utility of fisheye sky segmentation in urban climate studies. In particular, the Sky View Factor, a key variable for modeling radiative exchange and thermal comfort, can be estimated from hemispherical sky masks synthesized from Google Street View panoramas [34,35], enabling large-scale, low-cost assessments of urban morphology and sky openness. In parallel, forest ecology has leveraged fisheye sky segmentation to model light transmission under tree canopies. Hemispherical photographs have been used to estimate subcanopy shortwave radiation by analyzing canopy density and structure [36]. Despite the diversity of use cases, many public systems supporting these applications are geographically localized (often urban-only), and their masks can exhibit coarse sky–obstacle boundaries; such imperfections are inadequate for tasks that rely on precise skyline. Our work targets this gap by emphasizing pixel-wise sky–obstacle delineation in hemispherical views and by preserving thin occluders (e.g., branches, cables) needed for accurate sky openness, canopy light modeling, and visibility assessment.

These examples illustrate the central role of sky segmentation in modeling solar radiation exposure and sky openness in both natural and urban environments, and show that improving segmentation fidelity can provide tangible benefits across real-world applications. Building on this context, the next section describes the proposed fisheye-specific segmentation pipeline and its underlying methodology.

## 3. Materials and Methods

**Figure 1:**
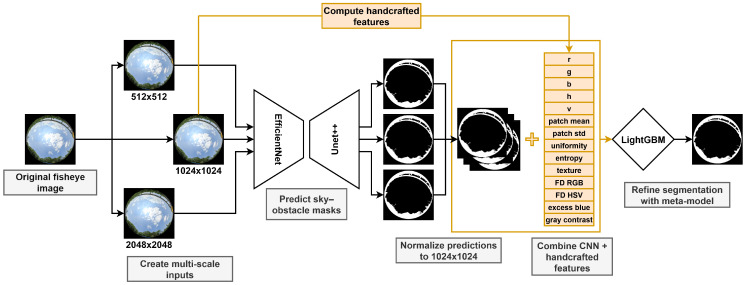
Overview of the proposed fisheye sky segmentation pipeline. Fisheye images are processed at multiple scales with a CNN, and refined by a LightGBM meta-model.

Figure 1 illustrates the main stages of the fisheye sky segmentation pipeline, from image preprocessing to model inference. Each input image is resized to three resolutions (512, 1024, and 2048 pixels), and a CNN is applied independently at each scale to produce probability maps. These predictions are then rescaled to a common 1024 × 1024 resolution and flattened for feature extraction. Together with handcrafted color, texture, and contrast descriptors, they serve as inputs to a Light Gradient Boosting Machine (LightGBM) [37], a GBDT meta-model that refines the segmentation and improves boundary accuracy across diverse scenes. The following subsections describe each stage of this pipeline in detail.

### 3.1. Dataset

#### 3.1.1. Data Generation Strategy

**Figure 2:**
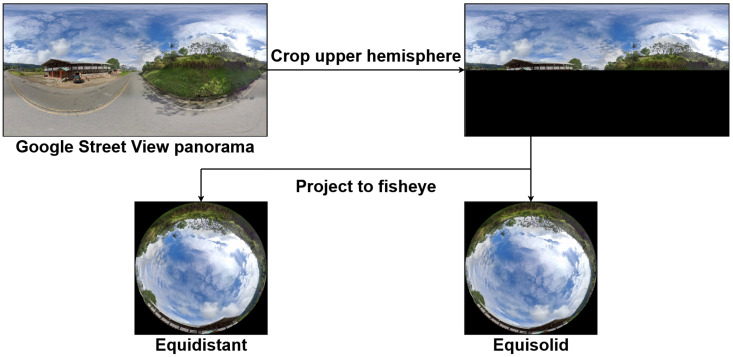
Conversion pipeline from a Google Street View panorama to synthetic fisheye views.

Given the absence of a suitable public benchmark that meets our criteria, we constructed a synthetic data generation pipeline based on Google Street View panoramas, illustrated in Figure 2. This pipeline, combined with annotation strategies, allows us to produce high-fidelity segmentation labels tailored specifically for global-scale sky analysis in fisheye imagery.

The panoramas provided by Google Street View are encoded in an equirectangular projection, where each pixel corresponds to geographic coordinates. The horizontal axis encodes the longitude λ∈[−π,π], and the vertical axis encodes the latitude $\varphi \in [-\frac{\pi }{2},\frac{\pi }{2}]$. To simulate a camera oriented toward the sky, we crop the top half of each panorama, corresponding to the upper hemisphere. The latitude range is therefore limited to $\varphi \in [0,\frac{\pi }{2}]$.

To simulate a fisheye image, we generate a normalized 2D grid of image coordinates $\left(x,y\right)\in {\left[-1,1\right]}^{2}$, representing a square sensor of size N×N. At each point in the grid, we compute the radial distance from the center:

(1) $$r=\sqrt{x^{2}+y^{2}}$$

We focus on the equidistant and equisolid-angle projections because they correspond to the most common models used in low-cost fisheye lenses [38].

##### Equidistant Projection

In the equidistant fisheye model, the incident angle θ between the optical axis and an incoming ray is linearly proportional to the radial distance *r* on the image plane, with *f* denoting the focal length of the projection [4]:

(2) $$r=f\times \theta \;\Rightarrow \;\theta =\frac{r}{f}$$

To map the upper hemisphere ($\theta \in [0,\frac{\pi }{2}]$) to the unit disk (r∈[0,1]), we set r=1 when $\theta =\frac{\pi }{2}$, which implies:

(3) $$f=\frac{1}{\pi /2}=\frac{2}{\pi }$$

Thus, the angle becomes

(4) $$\theta =r\times \frac{\pi }{2}$$

We compute spherical coordinates from each normalized fisheye pixel position. The zenith angle $\theta \in [0,\frac{\pi }{2}]$ corresponds to the incident angle defined by $\theta =r\times \frac{\pi }{2}$, while the azimuthal angle ϕ∈[−π,π] is computed as   

(5) ϕ=arctan2(y,x)

To sample RGB values from the source panorama, we project each computed spherical direction (ϕ,θ) to coordinates $\left({\mathrm{src}}_{x},{\mathrm{src}}_{y}\right)$ in the cropped equirectangular image, which has width *W* and height *H*. These coordinates are computed as

(6) $${\mathrm{src}}_{x}=\left(\frac{\phi }{2\pi }+\frac{1}{2}\right)\left(W-1\right)\;{\mathrm{src}}_{y}=\left(\frac{\theta }{\pi /2}\right)\left(H-1\right)$$

Here, ϕ∈[−π,π] and $\theta \in [0,\frac{\pi }{2}]$ are normalized to the range [0,1] before being scaled to pixel indices. $\left({\mathrm{src}}_{x},{\mathrm{src}}_{y}\right)$ then denote the corresponding pixel locations in the panorama. RGB values are sampled using nearest-neighbor interpolation. A circular mask is applied to exclude pixels where r>1, corresponding to rays outside the field of view.

##### Equisolid-Angle Projection

In the equisolid-angle projection model, the radial distance *r* from the image center is related to the incident angle θ [4]:

(7) $$r=2f\times \sin\left(\frac{\theta }{2}\right)$$

The inverse mapping recovers θ from *r*:

(8) $$\theta =2\times \arcsin\left(\frac{r}{2f}\right)$$

We normalize the projection such that the entire upper hemisphere ($\theta =\frac{\pi }{2}$) maps to the unit disk (r=1), yielding

(9) $$1=2f\times \sin\left(\frac{\pi }{4}\right)\;\Rightarrow \;f=\frac{1}{2\times \sin(\pi /4)}=\frac{1}{\sqrt{2}}$$

Substituting this into the inverse projection gives the final form

(10) $$\theta =2\times \arcsin\left(\frac{r}{\sqrt{2}}\right)$$

We then compute spherical coordinates as

(11) ϕ=arctan2(y,x)

where ϕ is the azimuthal angle and θ the zenith angle. The panorama indices $\left({\mathrm{src}}_{x},{\mathrm{src}}_{y}\right)$ are then computed using the same formulas as in the equidistant case. Nearest-neighbor and circular masking are applied identically.

This procedure enables the efficient synthesis of large volumes of realistic fisheye images using publicly available equirectangular panoramas. For our application, we prioritize rural and semi-urban scenes with diverse occlusions (e.g., trees, cables), while excluding night-time scenes and unoccluded landscapes. The resulting dataset covers a broad range of environmental conditions, including snow, desert, and mountainous terrain. Although nearest-neighbor interpolation is sufficient for segmentation purposes, bilinear interpolation could alternatively be used to produce smoother and more photorealistic fisheye renderings.

#### 3.1.2. Dataset Partitioning

Our dataset consists of both synthetic and real fisheye images. The synthetic images were generated from Google Street View panoramas using two distinct fisheye projection models. The CNN was trained on 80 synthetic images, including 63 generated with the equidistant projection and 17 with the equisolid-angle projection. The LightGBM meta-model was trained on an additional 60 synthetic images, consisting of 50 equidistant and 10 equisolid-angle samples.

To evaluate the performance of our models under real-world conditions, we additionally collected a set of 42 real fisheye images captured using two different smartphones: the Poco X4 Pro 5G (Poco/Xiaomi, Beijing, China) and the Samsung Galaxy A52s 5G (Samsung Electronics, Suwon, Republic of Korea). Both devices were equipped with 2 Pixter-branded fisheye lenses (Pixter, Paris, France) offering a 180° and 235° field of view, respectively. The images were acquired across three different sites: the CNRS station in Moulis (16 images), the LAAS-CNRS campus (16 images), and the University of Toulouse (10 images), thus covering a wide variety of rural, semi-urban, and urban settings. This diversity is critical for a robust evaluation of model generalization.

In total, the dataset comprises 182 fisheye images, which we partitioned into 80 training images for CNN learning, 60 images for meta-model refinement, 21 validation images, and 21 test images. The training and meta-model sets consist entirely of synthetic data, while the validation and test sets include only real fisheye images, ensuring that model evaluation is performed on authentic, unseen sensor data. The overall dataset composition, including projection type, quantity, and purpose, is summarized in Table 1.

#### 3.1.3. Annotation Strategy

Initial manual annotation. Coarse masks were first derived using simple image processing: Canny edge detection and intensity thresholding on the blue channel. The union of these masks provided an initial sky estimate, which was manually refined in GIMP [39]. This process produced 79 high-quality annotations (63 for training, 8 for validation, 8 for testing), each requiring approximately 40 min.

Semi-automated annotation. To accelerate dataset expansion, a preliminary segmentation model trained on the 63 manual annotations was used to pre-label additional fisheye images. Predictions were then corrected manually to achieve pixel-level accuracy. This strategy enabled efficient scaling to 103 more annotated images (60 for meta-model training, 17 for CNN training, 13 for validation, and 13 for testing).

Given the significant time required for pixel-level refinement, the dataset was not further expanded once additional annotations no longer yielded noticeable performance gains. With the final set of 182 annotated images, the segmentation framework achieves stable and competitive results.

### 3.2. Sky Segmentation Pipeline

#### 3.2.1. Fisheye Calibration and Disk Extraction

**Figure 3:**
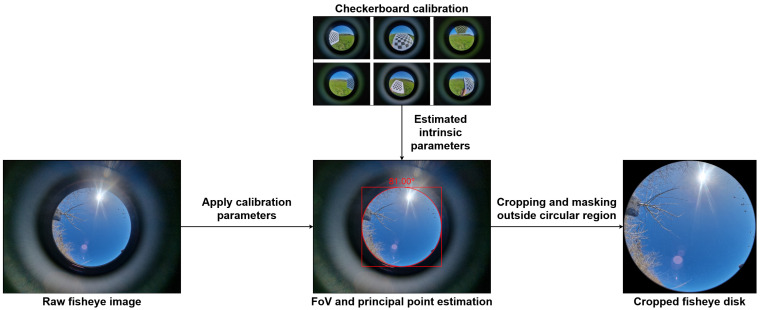
Fisheye calibration and disk extraction pipeline.

To obtain spatially accurate fisheye disk images, the camera is first calibrated using an enhanced omnidirectional calibration pipeline based on the method of Scaramuzza et al. [40], implemented via the py-omnicalib library [41]. Calibration is performed by detecting checkerboard corners across a diverse image set, refining subpixel correspondences, and optimizing the projection polynomial that maps 3D incident angles to radial image distances. From this calibration, the camera’s principal point and effective field of view (FoV) are estimated by minimizing the reprojection error in angular space. For consistency with hemispherical sky analysis, the estimated FoV is limited to $180^{\circ }$ (corresponding to a zenith angle range of 0–$90^{\circ }$). Consequently, even when the original fisheye lens exceeds this range, the image is cropped to retain only the upper hemisphere, ensuring a consistent representation of the sky dome. These intrinsic parameters allow computation of the maximum usable radius of the fisheye disk relative to the principal point. A square crop centered at the principal point is then applied, and all pixels outside the calculated circular region are masked, producing a clean, circularly bounded fisheye disk suitable for downstream pixel-wise processing, as illustrated in Figure 3. This calibration and disk-extraction procedure is applied to all real fisheye images used for validation and testing in our experiments.

#### 3.2.2. Data Augmentation Strategies

**Figure 4:**
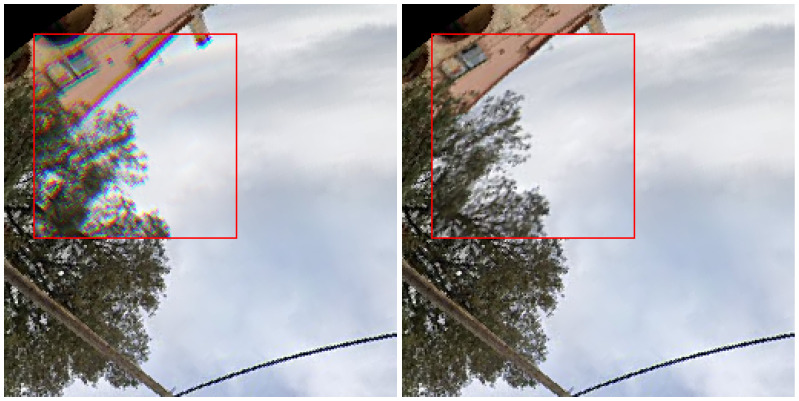
Examples of fisheye-specific augmentations. (**Left**) chromatic aberration simulation. (**Right**) synthetic radial distortion applied within the red square.

As discussed in Section 1.2, fisheye imagery introduces geometric and chromatic distortions that challenge conventional computer vision models. To improve robustness, we designed a data augmentation pipeline tailored to the optical characteristics of fisheye lenses (Figure 4). This pipeline combines two fisheye-specific augmentations with a set of standard photometric and structural transforms:

1. Chromatic Aberration: a common artifact in wide-angle optics, particularly in lower-cost fisheye lenses, where refractive dispersion causes different wavelengths of light to focus at varying depths. This leads to visible color fringing, often at object boundaries in peripheral image regions [5]. To simulate this effect (Figure 4, left), we implement a radial chromatic shift: the red and blue channels are displaced outward and inward, respectively, while the green channel remains fixed. The displacement scales with distance from the optical center, mimicking wavelength-dependent dispersion and training the network to handle color misalignments without overreliance on chromatic boundaries.
2. Fisheye Lens Distortion: to simulate the geometric variability of real-world fisheye optics, we apply synthetic radial distortion using OpenCV’s cv2.fisheye model [42]. We apply a parametric distortion defined by a sampled set of radial coefficients $D=[k_{1},k_{2},k_{3},k_{4}]$, alongside a camera matrix K derived from image dimensions. This transformation (Figure 4, right) introduces nonlinear warping consistent with lenses of varying focal lengths and manufacturing tolerances, exposing the model to a broad distribution of optical behaviors and improving generalization across domains.
3. Photometric and Structural Augmentations: to address variability in lighting and imaging conditions, we apply standard augmentations: random gamma, brightness, contrast, saturation, hue, and spatial filters such as Gaussian blur and sharpening to simulate optical softness or lens imperfections. We also include random horizontal and vertical flips, which preserve the circular symmetry of fisheye images while increasing orientation diversity in training. Additionally, we employ CutMix [43] to blend patches from different training images, increasing structural diversity and reducing overfitting to scene-specific layout.

In practice, the augmentation parameters were empirically tuned to improve generalization and limit overfitting. Photographic augmentations were applied with randomly sampled values reflecting weak augmentations, as these produced more stable results than strong ones. Fisheye-related augmentations were implemented as strong augmentations to expose the model to a wider range of distortions, and all augmentations were applied stochastically so that each training batch presented a different combination of transformations. Altogether, these augmentations addressed the core degradations found in fisheye imagery and, despite the relatively small dataset, enabled the training of a robust and efficient model capable of generalizing across diverse environments and lens geometries.

#### 3.2.3. Training Procedure

Model training was carried out on an NVIDIA H100 GPU with 80 GB VRAM using high-resolution input images of size 1024×1024, to achieve fine-grained pixel-level segmentation accuracy. The implementation was developed in Python 3.11.14 using PyTorch 2.6.0 with CUDA 12.6. The optimization process employed a combined loss function designed to balance pixel-wise classification and region overlap:

(12) L=α×BCELoss+(1−α)×DiceLoss,α=0.05

We used the AdamW optimizer with an initial learning rate of $4\times 10^{-4}$, weight decay of $1\times 10^{-3}$, and a cosine annealing learning rate schedule for gradual decay. To mitigate memory constraints while maintaining numerical stability, training was performed in mixed precision mode using the bfloat16 format. Due to the large input resolution and limited dataset size, the batch size was set to 4. To ensure reproducibility, a fixed seed was applied across all modules.

During training, we monitored the model using the training loss and the validation loss, the latter computed on a separate validation set. An early stopping criterion halted training after 4 consecutive epochs without improvement in the validation loss, and the corresponding loss curves were inspected to detect overfitting or training instability. After training, the final model selected according to the validation loss was evaluated on the independent test set using standard metrics, including Accuracy, Precision, Recall, F1 Score, and IoU. To ensure consistent evaluation across the valid image region, all metrics were computed exclusively within a circular binary mask corresponding to the fisheye projection area.

#### 3.2.4. Architecture Selection

To address the trade-offs between segmentation accuracy, computational cost, and generalization, we adopt an encoder–decoder framework for binary sky–obstacle segmentation. In this structure, the encoder extracts hierarchical feature representations from the fisheye input, while the decoder reconstructs the segmentation map at the original resolution. This design provides a well-established foundation for systematically benchmarking encoder backbones and decoder variants under controlled conditions.

We systematically benchmarked a wide range of pretrained encoder backbones commonly used in semantic segmentation, including ResNet-50, ResNet-101, ResNet-152 [44], InceptionV4 [45], and the EfficientNet [46] family from b0 to b7. All models were initialized using the AdvProp [47] pretraining strategy. Encoder comparisons were performed using a fixed decoder (U-Net++), and each configuration was trained and evaluated over 10 independent runs. Table 2 reports mean and standard deviation for region-level metrics (F1, IoU) and boundary-focused metrics (Boundary F1, Boundary IoU), which emphasize contour accuracy. The formal definitions of these metrics follow in Section 4.1.

Overall, these results highlight a clear hierarchy among encoder backbones. EfficientNet variants consistently outperform ResNet and InceptionV4 models across both region-level and boundary-focused metrics, while also using substantially fewer parameters. This trend is consistent with the design of EfficientNet: its compound scaling strategy increases depth, width, and input resolution together, which provides strong feature representations at several spatial scales. This is useful in our setting, where large, smooth sky regions coexist with thin, high-frequency sky–obstacle boundaries. The squeeze-and-excitation modules further adjust channel responses and may help distinguish small visual differences near the sky boundary. InceptionV4 uses parallel convolution branches and therefore tends to outperform ResNet, but it remains heavier and does not reach the boundary accuracy of mid-scale EfficientNet variants. ResNet, by contrast, mainly relies on depth scaling with less balanced changes in width and resolution, which leads to a less favorable trade-off between model size and accuracy and matches its lower boundary performance in our experiments.

Beyond the comparison across architecture families, we observe that increasing encoder capacity generally improves performance up to the mid-to-large EfficientNet variants. Among EfficientNet encoders, b4 through b7 form a plateau of top-performing models: they obtain the highest mean IoU and Boundary IoU, and their confidence intervals (mean ± standard deviation) largely overlap. Although EfficientNet-b5 exhibits a slightly lower mean IoU than EfficientNet-b4 in our benchmark, this difference is of the same order as the reported standard deviations and is therefore compatible with stochastic variability induced by random initialization, mini-batch sampling, and optimization dynamics. Given that the observed differences between EfficientNet-b4, b5, b6, and b7 are small relative to the experimental uncertainty, we treat these four variants as competitive candidates and subsequently evaluate all of them within our full segmentation + LGBM pipeline to assess how their final, post-processed performance differs.

In parallel, we evaluated several state-of-the-art decoder architectures: U-Net [48], U-Net++ [49], MA-Net [50], LinkNet [51], FPN [52], PSPNet [53], PAN [54], UPerNet [55], and SegFormer [56], while keeping the encoder fixed to EfficientNet-b7 for a strong and stable backbone. Decoder comparisons are summarized in Table 3.

**Figure 5:**
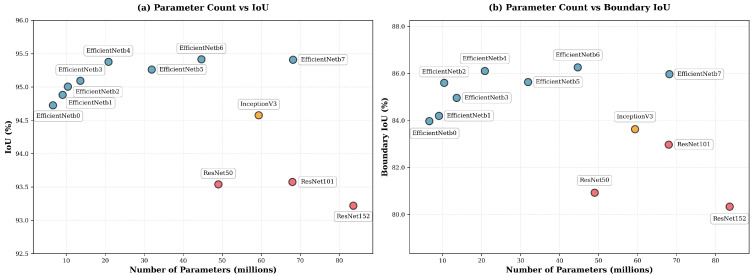
Encoder parameter count (**a**) vs. IoU and (**b**) Boundary IoU (decoder fixed to U-Net++).

**Figure 6:**
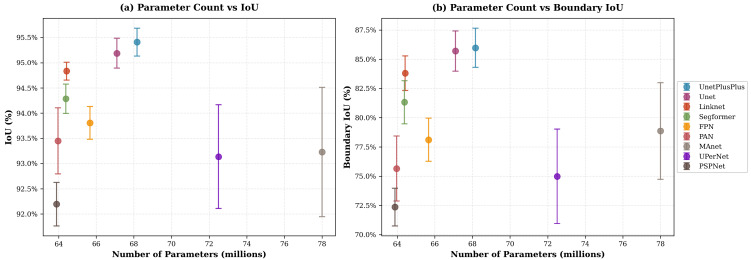
Decoder parameter count (**a**) vs. IoU and (**b**) Boundary IoU (encoder fixed to EfficientNet-b7).

The decoder comparison also reveals a consistent performance hierarchy. U-Net and U-Net++ achieve the strongest region-level IoU, with U-Net++ performing best overall. This trend is consistent with the design of U-Net++, whose nested dense skip connections and deep supervision promote repeated fusion of encoder features across multiple semantic depths. Such multi-scale aggregation is known to enhance gradient propagation and refine high-resolution details during reconstruction, which is particularly relevant for our task where skyline contours can be thin. LinkNet and SegFormer remain competitive in terms of region overlap but show weaker boundary fidelity, which may reflect the lighter skip-based fusion in LinkNet and the stronger global-context bias in SegFormer, both of which can be less favorable in contour-critical segmentation. Finally, pyramid-style decoders (FPN, PSPNet, UPerNet) lag behind in both IoU and boundary metrics; their emphasis on coarse contextual pooling is effective for broad scene parsing but is likely to attenuate fine-grained boundary recovery in this binary sky–obstacle setting. Finally, the two plots in Figure 5 and Figure 6 complement the results in Table 2 and Table 3 by illustrating the effect of model scaling. For encoders, EfficientNets form the Pareto frontier, achieving higher IoU and Boundary IoU at substantially lower parameter counts than ResNet or Inception backbones. For decoders, U-Net++ appears as the best compromise between complexity and accuracy, marginally heavier than U-Net but clearly superior in both region and boundary metrics. These visual trends align with our quantitative findings and motivate our final selection of EfficientNet-b4 to b7 paired with U-Net++.

#### 3.2.5. Post-Processing Meta-Model

To refine the pixel-level accuracy of our segmentation outputs, we introduce a supervised post-processing meta-model based on gradient-boosting decision trees, implemented with LightGBM.

From a modelling perspective, we adopt LightGBM for three reasons. First, it naturally operates at the pixel level and can jointly exploit heterogeneous feature types (CNN probabilities, color components, textural statistics) in a single model. Second, its non-linear decision structure is well suited to capturing interactions between multi-scale predictions and handcrafted cues, enabling it to refine ambiguous pixels that lie near the skyline or around thin occluders. Third, it offers an effective way to combine the complementary predictions obtained at 512×512, 1024×1024, and 2048×2048 resolutions. Rather than simply averaging or voting across scales, the LightGBM model learns, from the joint multi-scale probabilities and the handcrafted features, when a pixel behaves like a large homogeneous region (where lower-resolution predictions are more reliable) or like a fine-detail boundary (where higher-resolution predictions are preferred), and weighs the scale-specific probabilities accordingly, as qualitatively illustrated in Section 4.3.

**Figure 7:**
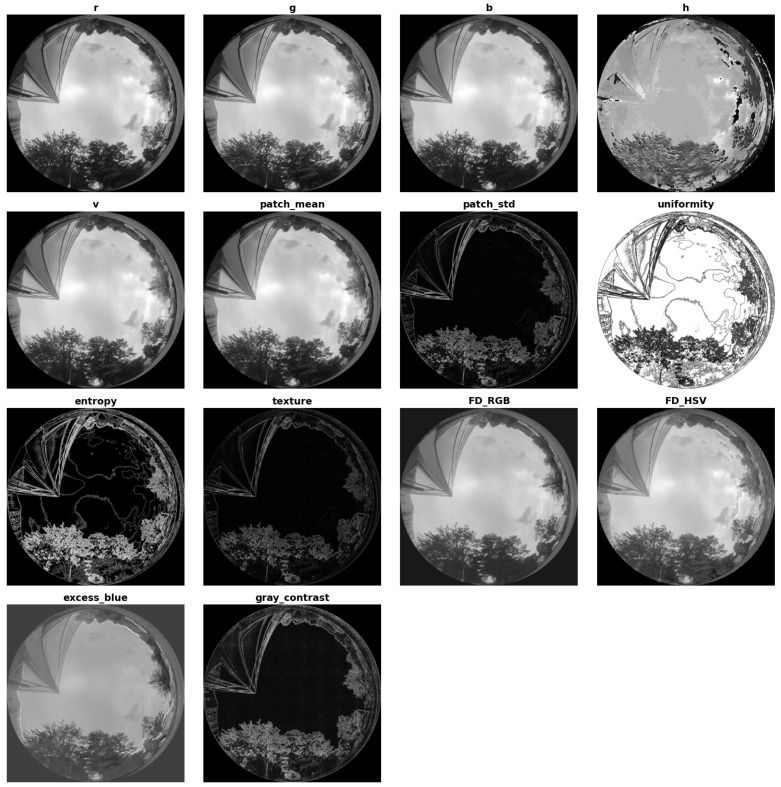
Representative feature maps corresponding to the descriptors used to train the LightGBM meta-model.

Specifically, LightGBM is trained on features extracted from a manually annotated dataset of 60 fisheye images, labeled using the semi-automated procedure described in Section 3.1.3. For each image, 50,000 pixels are randomly sampled within the circular fisheye region, resulting in 3,000,000 training instances. Each sampled pixel is represented by a comprehensive set of descriptors summarised in Table 4, with representative feature maps illustrated in Figure 7.

These descriptors combine multi-scale CNN probabilities with complementary color-based and statistical-structural features established in vision-based sky segmentation [22,23,35,57]. Multi-space color components (RGB, HSV, YCbCr) and chromatic indices such as dark channel, hue disparity, color saturation, and scene depth provide robust cues against radiometric and illumination variations [22,23]. Local statistical measures, including patch mean, standard deviation, third moment, smoothness, uniformity, entropy, texture, gradient magnitude, and contrast energy, capture both low-frequency homogeneity typical of sky regions and high-frequency heterogeneity characteristic of cluttered non-sky areas [23]. Empirical indices such as the Fisher Discriminant coefficients and the Excess Blue index further enhance discrimination of blue-dominant sky regions from diverse non-sky elements [35,57].

To reduce redundancy and model complexity, a two-stage feature selection process is applied. First, features with a Pearson correlation coefficient below 0.5 with respect to the ground-truth labels are discarded. Then, highly collinear variables are removed while retaining the CNN-derived multi-scale predictions due to their strong predictive value. The final selected set therefore includes the CNN probability maps at 512, 1024, and 2048 input resolutions, together with selected color and textural descriptors (e.g., r, g, b, h, v, uniformity, entropy, texture, Fisher Discriminant features), the excess blue index, and the grayscale contrast energy.

In practice, this meta-model acts as a pixel-wise refinement layer, particularly effective in sparse or occluded scenes where the sky is partially obstructed by small objects and where a single-resolution CNN model tends to underperform.

## 4. Results

### 4.1. Model Evaluation

To evaluate the final segmentation performance, we trained U-Net++ models with EfficientNet-b4, b5, b6, and b7 encoders, corresponding to the top-performing backbones identified in the architecture benchmark. Model performance was assessed using the F1 score and IoU, computed as weighted averages by class support. In addition, we report two boundary-focused metrics: Boundary IoU [58] and Boundary F1 [59]. Boundary IoU evaluates overlap only within narrow bands around the predicted and ground-truth contours, yielding a boundary-sensitive analogue of IoU that is symmetric and less biased by region interiors; we follow the reference implementation and use a dilation ratio of 0.02. Boundary F1 (also called the boundary F-measure) is the harmonic mean of boundary precision and recall under a small localization tolerance; throughout this work we use a tolerance of τ=3 pixels. These complementary measures directly target skyline fidelity and contour alignment. Baseline CNN-only results are summarized in Table 5.

For each encoder, we further trained a dedicated post-processing meta-model based on LightGBM, as described in Section 3.2.5. Hyperparameter tuning was conducted using Optuna [60]. During training, we employed early stopping on the validation metric to automatically select the effective number of boosting iterations (trees). In practice, early stopping consistently halted training after fewer than 100 trees across all configurations, resulting in compact boosted tree models and keeping the post-processing stage lightweight. To convert the probabilistic output of the LGBM model into a binary mask, we evaluated several thresholding strategies: simple fixed thresholding, Otsu’s method [61], and Conditional Random Fields (CRF) [62]. Empirically, a single fixed decision threshold tuned on the validation set yielded the best overall results for all encoders. The results obtained with LGBM post-processing are summarized in Table 6.

The LGBM post-processing module yields consistent improvements over the CNN-only baselines for all EfficientNet variants, enhancing both region-level and boundary-focused metrics. Across encoders, IoU increases by roughly 0.8–0.9%, while Boundary IoU and Boundary F1 improve by up to about 3.4% and 5.2%, respectively. Although these absolute gains may appear modest, on 1024 × 1024 images they correspond to correcting misclassified labels over thousands of pixels per frame, predominantly along object boundaries, which is perceptually significant for skyline delineation. Among the refined models, EfficientNet-b7 attains the highest region-level F1, IoU, and Boundary F1, whereas EfficientNet-b6 slightly outperforms it on Boundary IoU. EfficientNet-b4 closely matches b5 in region metrics but remains noticeably weaker on boundary scores, reinforcing the importance of explicitly evaluating boundary-sensitive criteria. In practice, this results in a clear accuracy–efficiency trade-off: EfficientNet-b7 maximizes segmentation quality, while smaller variants (b4–b6) sacrifice only a small amount of performance in exchange for substantially fewer parameters and more lightweight inference. Overall, combining a deep segmentation backbone with a supervised refinement layer yields precise sky–obstacle delineation with improved region overlap and markedly enhanced skyline fidelity across a range of model capacities.

**Figure 8:**
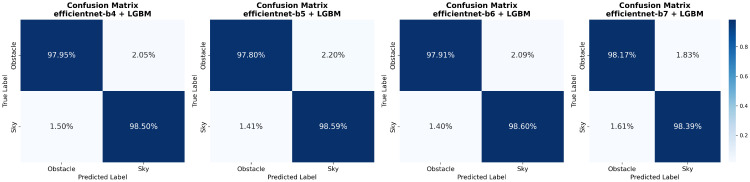
Confusion matrices for the LGBM-refined models on the test set, showing class-wise sky/obstacle prediction rates. From left to right: EfficientNet-b4, EfficientNet-b5, EfficientNet-b6, and EfficientNet-b7 encoders.

In addition to the region-level and boundary-focused metrics, we also examine confusion matrices for the LGBM-refined models, shown in Figure 8. These matrices provide a class-wise view of prediction behaviour by separating errors into false-sky (obstacle pixels predicted as sky) and false-obstacle (sky pixels predicted as obstacles). For solar irradiance forecasting, a pessimistic model is preferable: it should be more inclined to label ambiguous pixels as obstacles rather than sky, so that irradiance is slightly underestimated rather than overestimated. Among the tested variants, the LGBM + EfficientNet-b7 model best matches this behaviour, achieving the lowest false-sky rate while accepting a slightly higher false-obstacle rate compared to the other architectures.

**Figure 9:**
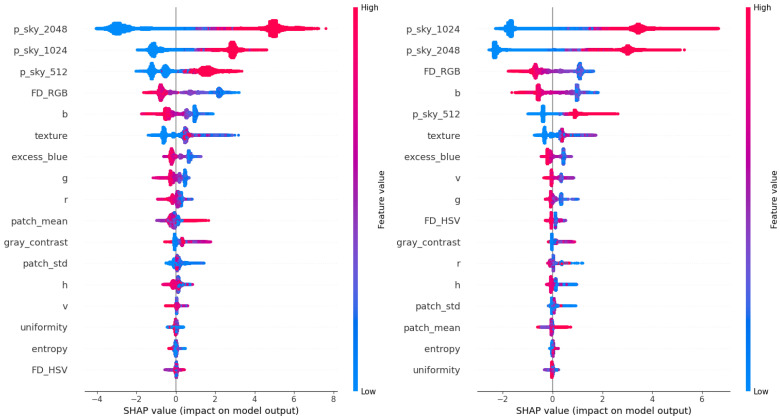
SHAP summary plots for the LightGBM models based on EfficientNet-b5 predictions (**left**) and EfficientNet-b7 predictions (**right**). Each dot corresponds to a pixel sample, colored by feature value; greater horizontal spread indicates higher predictive influence.

To better understand which features drive the improved segmentation accuracy, we analyzed representative LightGBM meta-models using SHAP [63]. We focus here on the EfficientNet-b5 and EfficientNet-b7 backbones, which span mid to high-capacity encoders. Figure 9 shows that the most impactful features across both configurations include multi-scale CNN predictions (p_sky_2048, p_sky_1024, p_sky_512), along with handcrafted descriptors such as Fisher Discriminant in RGB space, blue channel intensity, and texture. This highlights the value of combining deep and handcrafted features for reliable sky–obstacle boundary refinement, especially across varying spatial resolutions.

**Figure 10:**
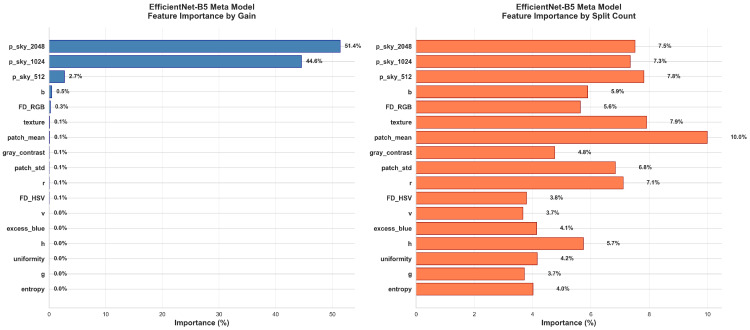

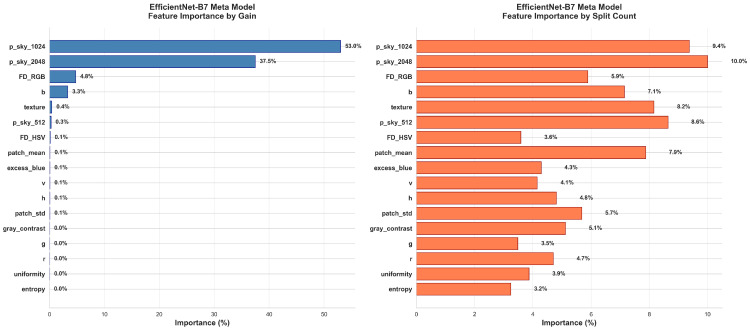
Built-in LightGBM feature importances for EfficientNet-b5 (**top**) and EfficientNet-b7 (**bottom**) meta-models, evaluated using gain (**left**) and split count (**right**).

To complement the SHAP-based interpretation, the built-in LightGBM feature importances were also examined using the gain and split count criteria (Figure 10) for the same two representative configurations. The gain metric measures how much each feature contributes to the reduction of the loss function, whereas the split count reflects how frequently a feature is selected across all decision trees.

In both EfficientNet-b5 and b7 meta-models, the multi-scale CNN predictions dominate the gain ranking, confirming that the base segmentation probabilities are the most informative predictors for sky–obstacle discrimination. However, the split count analysis reveals a broader distribution, with local statistical, textural, and color-based features appearing more frequently in splits. These features provide complementary thresholds that support fine-grained boundary refinement, even if their overall contribution remains smaller.

Minor differences between gain and split rankings mainly arise from correlations among color and luminance-related descriptors, reflecting mild multicollinearity effects also observed in the SHAP analysis. Overall, both analyses confirm that the LightGBM meta-model primarily relies on multi-scale CNN-derived probabilities while leveraging handcrafted features to adjust decision boundaries in visually complex regions.

### 4.2. Impact of the New Augmentations on Model Performance

To evaluate the contribution of chromatic aberration and fisheye lens distortion augmentations, we conducted an ablation study where both were omitted. EfficientNet-b5 and EfficientNet-b7 encoders with a U-Net++ decoder were trained following the same protocol described in Section 3.2.4, and each configuration was repeated across 10 independent runs. We report the mean IoU and standard deviation to account for variability.

When trained without the augmentations, EfficientNet-b5 achieved a mean IoU of 95.17±0.71, compared to 95.31±0.42 with augmentations. For EfficientNet-b7, the mean IoU dropped from 95.37±0.62 (with augmentations) to 95.23±0.57 (without augmentations).

Although the numerical differences are modest, these results indicate that the proposed augmentations provide measurable benefits. More importantly, they enhance robustness by exposing models to optical variability, thereby supporting improved generalization across diverse lenses and imaging conditions. This is particularly valuable for real-world deployment, where heterogeneous hardware and optical characteristics are common.

### 4.3. Qualitative Analysis of Model Predictions

**Figure 11:**
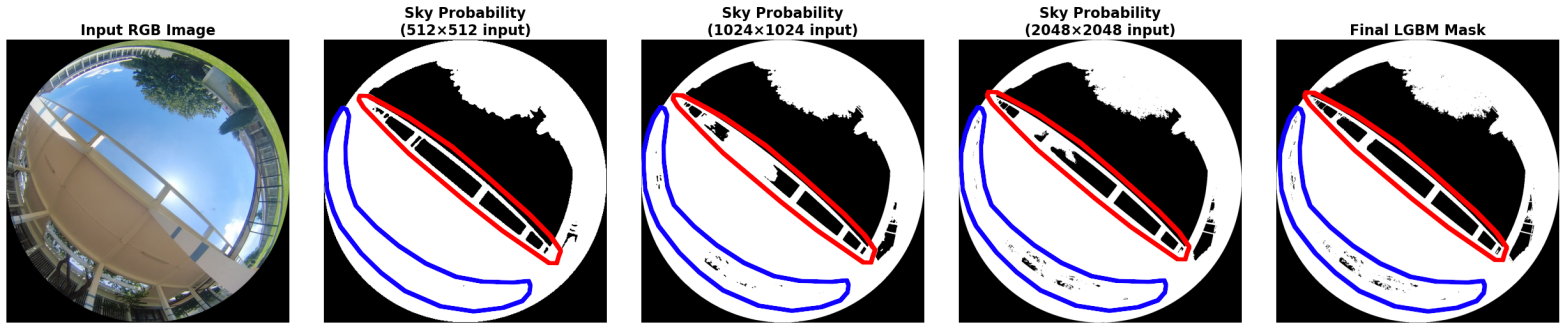
Visualization of prediction complementarity on a challenging validation image. From left to right: input RGB fisheye image, EfficientNet-b5 sky–obstacle probability maps for input resolutions of 512 × 512, 1024 × 1024, and 2048 × 2048 pixels, and final binary mask produced by the LightGBM meta-model. The scene includes a complex skyline, with an obstacle dividing the sky into separate regions (red area) and dense vegetation in the lower hemisphere (blue area). The original image resolution is 1024 × 1024 pixels.

Figure 11 illustrates the advantage of using a LightGBM meta-model to enhance segmentation predictions. At 1024×1024 resolution, a segmentation error is visible in the red-highlighted area, where a section of the sky is occluded by an overhead obstacle. Interestingly, this error is absent in the 512×512 prediction, which better captures large structural context but suffers from coarser boundary precision, as seen in the blue-highlighted region. In contrast, the 2048×2048 prediction preserves fine details but can be less stable in large homogeneous regions. The LightGBM model effectively fuses the strengths of predictions at different resolutions, producing a refined output that minimizes both types of error.

**Figure 12:**
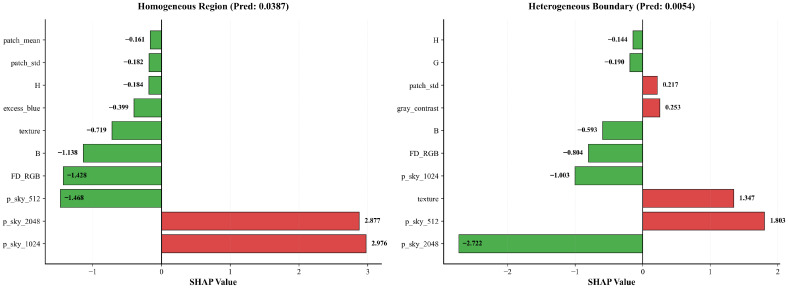
Local SHAP waterfall plots showing the top 10 features (with highest SHAP contribution) for two ground-truth sky pixels from Figure 11. (**Left plot**) sky pixel in a homogeneous region. (**Right plot**) sky pixel on a heterogeneous sky–obstacle boundary.

To better understand how the meta-model exploits these complementary behaviours, Figure 12 reports local SHAP waterfall plots for two representative sky pixels taken from the red and blue areas in Figure 11. In both cases, the desired output is a probability close to zero, corresponding to the sky class. For the pixel located in a homogeneous sky region (red area, left panel), the prediction values at 1024×1024 and 2048×2048 strongly push the model towards the obstacle class, but this tendency is counteracted by the prediction at 512×512 together with several handcrafted descriptors (blue-channel intensity, Fisher Discriminant, texture, Excess Blue, and local statistics), which contribute in the opposite direction and keep the final probability close to zero. For the pixel on a heterogeneous boundary (blue area, right panel), the situation is reversed: the coarse prediction at 512×512 and the local texture favour the obstacle class, while the finer-scale prediction at 2048×2048 and 1024×1024, along with Fisher Discriminant and other colour features, pulls the score back towards the sky class.

Formally, the LightGBM model outputs a logit value f(x) for each pixel, which is decomposed by SHAP as

(13) $$f\left(x\right)=E\left[f\left(x\right)\right]+\sum_{i}\phi_{i},$$

where E[f(x)] is the global base value (the expected logit over the training data) and $\phi_{i}$ is the contribution of feature *i* for that pixel. The corresponding obstacle probability is obtained by applying the sigmoid function

(14) $$p=\sigma \left(f(x)\right)=\frac{1}{1+\exp\left(-f(x)\right)}.$$

For the homogeneous-sky pixel, the base value is E[f(x)]=−3.2300 and the sum of all SHAP contributions is $\sum_{i}\phi_{i}=0.0186$. The raw logit is therefore

(15) f(x)=−3.2300+0.0186=−3.2114,

and the obstacle probability becomes

(16) $$p=\frac{1}{1+\exp\left(-(-3.2114)\right)}\approx 0.0387.$$

For the boundary sky pixel, the same base value combines with a SHAP sum of $\sum_{i}\phi_{i}=-1.9775$, giving

(17) f(x)=−3.2300+(−1.9775)=−5.2075,

and thus

(18) $$p=\frac{1}{1+\exp\left(-(-5.2075)\right)}\approx 0.0054.$$

These examples illustrate how the meta-model adaptively reweights the multi-scale prediction values and handcrafted features so that, even when one scale misclassifies a pixel, the final decision remains consistent with the sky label.

This multi-scale fusion strategy proves especially useful in heterogeneous environments: low-resolution predictions (e.g., 512 × 512) improve structural coherence in urban settings with frequent occlusions, while high-resolution predictions (e.g., 2048 × 2048) preserve fine details important in rural or vegetated landscapes.

**Figure 13:**
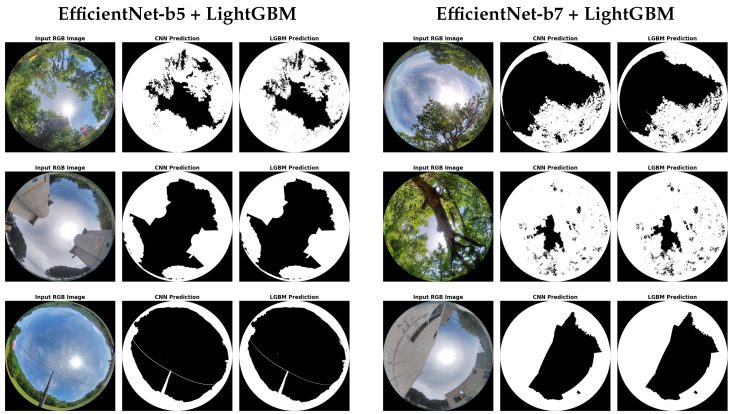
Qualitative sky–obstacle segmentation on unseen fisheye validation and test images. The left panel shows results from EfficientNet-b5 and the right panel from EfficientNet-b7. Each panel shows, from left to right, the input RGB image, the CNN prediction, and the LightGBM-refined mask.

Figure 13 presents additional qualitative results for the EfficientNet-b5 and EfficientNet-b7 models across a variety of urban and rural scenes. Both models demonstrate strong generalization across environments, with the LGBM post-processing consistently correcting local errors and improving edge coherence. These visualizations support the quantitative performance gains reported in Section 4.3, highlighting the practical robustness of the combined segmentation pipeline in real-world scenarios.

### 4.4. Comparison with an External Baseline

As highlighted in Section 1.3, direct comparison with existing segmentation benchmarks is hindered by the scarcity of high-quality fisheye datasets with accurate sky annotations. However, we identified a recent work [64] that employs a similar methodology using equirectangular panoramas from Google Street View. This study introduces the CVRG-Pano dataset and proposes two U-Net-based architectures for semantic segmentation of panoramic imagery.

**Figure 14:**
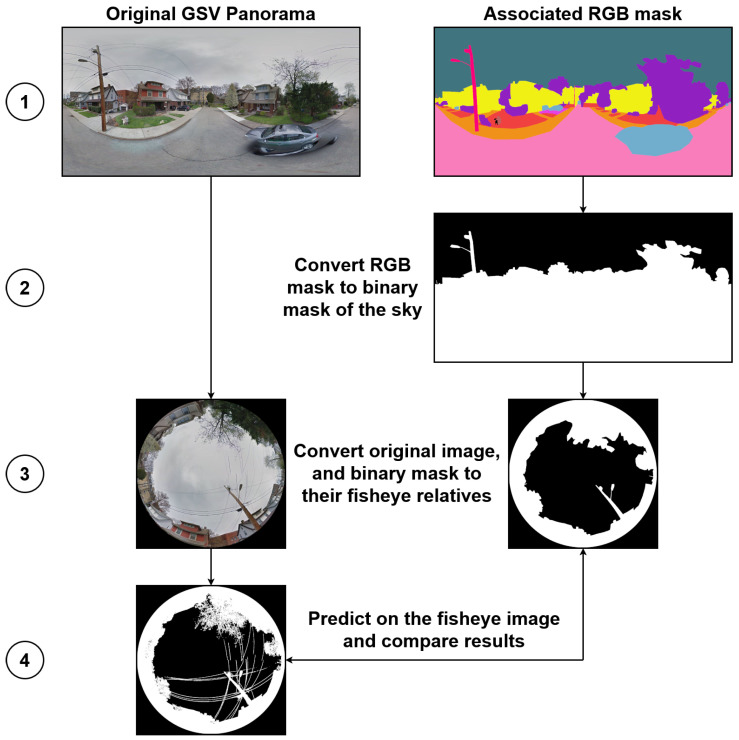
Evaluation pipeline showing the four steps used to assess model generalization on the CVRG-Pano dataset. A detailed description of the four steps is provided in the main text.

To enable a fair comparison, we employed our fisheye transformation pipeline to convert the CVRG-Pano test images and their associated multi-class masks into the fisheye domain. The evaluation procedure is summarized in Figure 14. In step 1, each panoramic image is paired with its ground-truth semantic mask. In step 2, the RGB mask is converted to a binary sky mask. Step 3 involves projecting both the RGB image and the binary sky mask into the fisheye domain using the equidistant projection model, which is representative of low-cost commercial fisheye lenses. Finally, in step 4, we perform sky segmentation using our trained U-Net++ models with EfficientNet-b4, b5, b6, and b7 backbones, each evaluated with and without LightGBM-based post-processing refinement.

We report IoU scores in Table 7. Despite annotation inconsistencies in CVRG-Pano, such as coarse boundaries and the omission of fine-grained obstacles like cables and thin branches, our models maintain strong generalization and achieve high performance, even on data not tailored for precise sky–obstacle segmentation.

The IoU values of the base models on CVRG-Pano closely match those obtained on our internal fisheye test set, indicating good cross-dataset generalization. In contrast to our own dataset, LightGBM post-processing does not systematically improve IoU here: three backbones (b4–b6) exhibit a slight decrease, while EfficientNet-b7 shows only a modest gain. This behaviour is consistent with the nature of the CVRG-Pano annotations, whose coarse boundaries and missing small obstacles penalize boundary-focused refinements that produce sharper, more detailed skylines than those represented in the ground truth. In other words, the meta-model tends to correct visually plausible fine structures (e.g., tree crowns, cables) that are not labeled, which can reduce IoU with respect to the provided masks.

Nevertheless, all configurations achieve IoUs around 95–96% on this external dataset, underscoring the robustness of the proposed pipeline. From a practical perspective, the LightGBM-refined predictions may be particularly valuable not for maximizing IoU on CVRG-Pano, but for upgrading the quality of its sky annotations by supplying more accurate, high-resolution sky–obstacle boundaries.

### 4.5. End-to-End Runtime Performance on CPU and GPU

We evaluated the runtime of the main components of the segmentation pipeline for EfficientNet-b4, b5, b6, and b7 backbones. For each model, we measured the total time required for all CNN forward passes across the three input resolutions used in our pipeline (512 × 512, 1024 × 1024, and 2048 × 2048), together with the time spent on handcrafted feature extraction and subsequent LightGBM inference. All timings are averaged over 42 fisheye images of size 1024 × 1024. CPU experiments were performed on an Intel Core i5-1335U (1.3 GHz). For GPU measurements, CNN inference was executed on an NVIDIA H100 (80 GB VRAM) hosted in a server with an AMD EPYC 9124 (3 GHz), while feature extraction and LightGBM inference remained CPU-bound. The full set of results is reported in Table 8.

On the CPU-only configuration, the end-to-end latency ranges from 16.8 s (EfficientNet-b4) to 30.7 s (EfficientNet-b7). The CNN stage accounts for more than 90% of this cost and is itself dominated by the 2048 × 2048 forward pass, which represents roughly 70–80% of the total CNN runtime. For comparison, a single 1024 × 1024 CNN forward pass requires only 2.8–5.6 s on CPU, so the complete multi-scale + LightGBM pipeline is approximately 5–6× slower than a single-resolution prediction. This highlights the computational price of the improved accuracy obtained with multi-scale inference and post-processing.

On the GPU configuration, multi-scale CNN inference becomes inexpensive, with total forward times between 0.33 s and 0.50 s depending on the backbone. A single 1024 × 1024 CNN pass on the H100 requires only 0.06–0.12 s, illustrating the lower bound achievable on high-end accelerators. However, the overall runtime (2.4–2.8 s) is dominated by CPU-side feature extraction and LightGBM inference, whose performance depends primarily on the host CPU rather than on the GPU. As a result, differences between backbones shrink to only a few hundred milliseconds, making the most accurate model (EfficientNet-b7) essentially cost-neutral on GPU. In contrast, on CPU-only systems, smaller backbones such as b4 or b5 offer a more favorable accuracy–latency trade-off while retaining strong segmentation performance.

## 5. Discussion

This study demonstrates that sky–obstacle segmentation in hemispherical fisheye imagery can be effectively achieved through a hybrid framework combining deep learning with structured post-processing. Synthetic fisheye views were generated from Google Street View panoramas and enriched with lens and lighting-aware augmentations to produce a dataset that captures global variability without the cost of large-scale field acquisition. Experimental results confirm that convolutional neural networks trained on this dataset achieve high accuracy on real fisheye images, and that the LightGBM refinement step further improves segmentation precision along challenging boundaries, particularly when evaluated with boundary-focused metrics.

Two aspects are particularly noteworthy. First, the proposed augmentations not only yield gains in IoU but also improve robustness. This is especially valuable given the relatively limited dataset size: augmentations compensate for data scarcity by exposing the model to a wider range of optical variability. As a result, the framework generalizes well to real-world fisheye captures and to an independent public benchmark (CVRG-Pano), underscoring its reliability beyond the training distribution. Second, the integration of the post-processing meta-model enables a true pixel-wise analysis. Leveraging CNN-derived multi-scale predictions alongside handcrafted descriptors, the meta-model refines fine occlusions such as vegetation or cables. This precise pixel-wise fidelity is particularly important for long-term solar resource assessment, where shading factor estimation depends on accurate delineation of skyline masks.

Beyond these quantitative gains, the behaviour of the hybrid CNN-LightGBM pipeline is qualitatively different from more classical graph-based post-processing strategies, such as dense CRFs. These models typically enforce local smoothness with contrast-sensitive pairwise terms, which is effective at removing small isolated errors but tends to oversmooth thin occluders and assumes that boundaries align well with colour or intensity edges. In our fisheye setting, thin structures (branches, cables) and chromatic aberrations frequently violate these assumptions. By contrast, the LightGBM meta-model operates directly on heterogeneous, pixel-wise features: multi-scale CNN probabilities, multi-space colour components, local statistics and texture descriptors, and learns non-linear combinations that distinguish large homogeneous sky regions from cluttered boundaries. This design explains why the hybrid model yields not only modest improvements in region-level IoU over the pure CNN baseline, but markedly larger gains on boundary-sensitive metrics (Boundary IoU and Boundary F1). An additional advantage of the LightGBM layer is its compatibility with post-hoc interpretability methods: gain and split-based feature importances, together with SHAP values, show that the meta-model primarily relies on multi-scale CNN probabilities while using colour and textural descriptors to refine decisions in visually complex regions. Together with these feature-attribution results, the observed performance gains suggest that most of the useful spatial regularisation is already captured by the learned meta-model through its access to multi-scale CNN predictions and local contextual descriptors, making the hybrid CNN-LightGBM approach a data-driven alternative to manually designed CRF and graph-based post-processing in fisheye sky imagery.

### 5.1. Limitations

While the proposed framework is effective, several limitations should be acknowledged. Our pipeline includes a calibration-based procedure for fisheye disk extraction (Section 3.2.1), which requires checkerboard calibration images. Alternative approaches using image processing to automatically crop the fisheye disk without calibration do exist, but were not implemented here, since our calibration-driven method was sufficient in the present context.

In addition, the full multi-scale pipeline is computationally demanding, especially on low-power CPUs. Most of the runtime is spent in the repeated CNN forward passes at high resolutions (in particular 2048 × 2048), with feature extraction and LightGBM inference adding a smaller but non-negligible overhead. As a result, the current design is not specifically tailored to ultra-constrained embedded platforms. In scenarios where efficiency is paramount, practitioners can trade accuracy for speed in several ways: by adopting lighter encoders (e.g., EfficientNet-b0/b1 with U-Net or U-Net++), by reducing the number or the maximum scale of input resolutions (for instance, dropping the 2048 × 2048 pass or relying on a single 1024 × 1024 prediction), and by limiting the set of handcrafted features used by the meta-model. These variants provide straightforward paths to substantially lower latency while retaining a large fraction of the segmentation quality.

Finally, the dataset size remains modest relative to large-scale vision benchmarks. Although augmentation strategies mitigate this limitation, broader geographic and temporal diversity would further strengthen robustness. In particular, the dataset was originally collected for solar irradiance prediction, and therefore contains very few low-illumination conditions (e.g., night-time scenes). The distribution of test images could also be improved: while it already spans urban, semi-urban, and rural areas, it lacks a wider range of environmental conditions such as snow, fog, or heavy rain, which are important for fully stress-testing sky–obstacle segmentation models.

### 5.2. Future Work

Potential extensions concern both modeling and data. On the methodological side, a promising direction is the design of distortion-aware architectures tailored to fisheye sky segmentation. Integrating operators such as deformable convolutions or radial/polar transformers could better account for the non-uniform spatial sampling of fisheye lenses; to the best of our knowledge, no dedicated distortion-aware framework has yet been proposed specifically for this task.

At the data generation stage, our current fisheye rendering pipeline relies on nearest-neighbor interpolation when mapping equirectangular panoramas to the fisheye domain. While this choice is sufficient for segmentation and preserves label alignment, it can introduce blocky artefacts in the synthesized images. Future work could investigate higher-order resampling schemes, such as bilinear or bicubic interpolation, or learnable warping modules to produce smoother, more photorealistic fisheye renderings, which may further improve generalization to real-world imagery.

From a data perspective, applications that go beyond solar irradiance prediction may benefit from broader coverage than offered by the present dataset. In particular, additional fisheye data could target low-illumination scenarios and a wider range of weather and environmental conditions, as well as more diverse geographic and seasonal contexts.

## 6. Conclusions

A scalable and affordable framework for hemispherical sky–obstacle segmentation has been presented, combining synthetic fisheye data generation from Google Street View panoramas, lens-aware augmentation, and a hybrid CNN-LightGBM architecture. To ensure transparency and reproducibility, the complete source code and the dataset annotations are publicly released, together with a script that enables retrieval of the original panoramas via the official Google Street View API. The real fisheye images captured with smartphone lens attachments, used for validation and testing, are also shared in full.

On real fisheye images acquired with low-cost smartphone systems, the proposed framework achieves up to 96.63% IoU, 98.29% F1, 92.25% Boundary F1, and 91.43% Boundary IoU, demonstrating both high region-level accuracy and precise sky–obstacle boundary delineation across devices and acquisition sites. Additional experiments on an external panoramic benchmark (CVRG-Pano) confirm that these models generalize well beyond the training distribution, even when evaluated on data not specifically tailored for fine-grained skyline annotations.

Beyond its quantitative performance, the framework establishes a practical basis for long-term solar resource assessment, while remaining relevant for environmental monitoring and navigation tasks. The complete segmentation pipeline is integrated into a shading estimation tool that computes shading factors from fisheye imagery, enabling reliable quantification of obstacle-induced solar losses along the sun’s path.

Future extensions may further improve the approach through distortion-aware architectures tailored to fisheye optics, lightweight variants suitable for constrained hardware, and expanded datasets that cover a broader range of geographic contexts, illumination regimes, and weather conditions to strengthen generalization.

## Figures and Tables

**Table 1:** Summary of dataset composition by projection model, quantity, and purpose.

Data Type	Projection Model	Quantity	Purpose	Source
Synthetic	Equidistant	63	CNN training	Google Street View
Synthetic	Equisolid-angle	17	CNN training	Google Street View
Synthetic	Equidistant	50	Meta-model training	Google Street View
Synthetic	Equisolid-angle	10	Meta-model training	Google Street View
Real	–	21	Validation	Smartphone captures
Real	–	21	Test	Smartphone captures
Total		182		

**Table 2:** Performance comparison of different encoders using a U-Net++ decoder. Metrics are reported in %. Bold values indicate the best result within each column.

Encoder	Params (M)	F1	IoU	Boundary F1	Boundary IoU	Time/Epoch (s)
ResNet-50	48.99	96.66 ± 0.55	93.54 ± 1.02	76.27 ± 7.11	80.92 ± 3.68	11.00 ± 0.49
ResNet-101	67.98	96.68 ± 0.56	93.58 ± 1.04	76.90 ± 5.95	82.97 ± 3.40	11.00 ± 0.49
ResNet-152	83.62	96.49 ± 0.55	93.22 ± 1.02	74.31 ± 6.03	80.33 ± 2.96	12.00 ± 0.64
InceptionV4	59.36	97.21 ± 0.38	94.58 ± 0.72	81.21 ± 4.51	83.63 ± 2.72	12.00 ± 0.00
EfficientNet-b0	**6.57**	97.29 ± 0.14	94.72 ± 0.27	79.93 ± 4.20	83.97 ± 1.88	**9.00 ± 0.00**
EfficientNet-b1	9.08	97.37 ± 0.15	94.88 ± 0.28	80.81 ± 3.26	84.19 ± 1.62	9.00 ± 0.46
EfficientNet-b2	10.40	97.44 ± 0.18	95.01 ± 0.33	83.13 ± 2.75	85.60 ± 1.71	9.00 ± 0.40
EfficientNet-b3	13.62	97.48 ± 0.16	95.09 ± 0.31	81.89 ± 4.33	84.96 ± 1.99	10.00 ± 0.00
EfficientNet-b4	20.81	97.63 ± 0.13	95.38 ± 0.26	**84.88 ± 1.89**	86.11 ± 1.54	11.00 ± 0.00
EfficientNet-b5	31.91	97.57 ± 0.16	95.26 ± 0.30	84.14 ± 2.80	85.63 ± 1.71	12.00 ± 0.46
EfficientNet-b6	44.67	**97.65 ± 0.14**	**95.42 ± 0.27**	84.70 ± 3.27	**86.26 ± 1.98**	14.00 ± 0.00
EfficientNet-b7	68.16	**97.65 ± 0.15**	95.41 ± 0.28	84.41 ± 2.93	85.97 ± 1.68	16.00 ± 0.43

**Table 3:** Performance comparison of different decoders using an EfficientNet-b7 encoder. Metrics are reported in %. Bold values indicate the best result within each column.

Decoder	Params (M)	F1	IoU	Boundary F1	Boundary IoU	Time/Epoch (s)
U-Net	67.10	97.53 ± 0.16	95.19 ± 0.30	83.14 ± 2.02	85.69 ± 1.72	14.00 ± 0.40
U-Net++	68.16	**97.65 ± 0.15**	**95.41 ± 0.28**	**84.41 ± 2.93**	**85.97 ± 1.68**	16.00 ± 0.43
MA-Net	78.00	96.49 ± 0.69	93.23 ± 1.28	71.46 ± 6.96	78.86 ± 4.13	14.00 ± 0.00
LinkNet	64.42	97.35 ± 0.09	94.83 ± 0.18	78.96 ± 2.47	83.80 ± 1.48	13.00 ± 0.49
FPN	65.67	96.80 ± 0.17	93.81 ± 0.32	70.64 ± 4.55	78.11 ± 1.85	12.00 ± 0.00
PSPNet	**63.88**	95.94 ± 0.23	92.19 ± 0.43	56.50 ± 2.59	72.36 ± 1.61	**10.00 ± 0.00**
PAN	63.96	96.61 ± 0.35	93.45 ± 0.66	64.18 ± 5.26	75.65 ± 2.77	34.00 ± 0.30
UPerNet	72.50	96.44 ± 0.55	93.14 ± 1.03	63.23 ± 8.24	74.99 ± 4.03	13.00 ± 0.00
SegFormer	64.39	97.06 ± 0.15	94.29 ± 0.29	77.35 ± 2.77	81.32 ± 1.84	12.00 ± 0.43

**Table 4:** Summary of all pixel-wise features extracted for LightGBM training. Features shown in bold were retained in the final meta-model after feature selection.

Name	Symbol	Computation
**Sky prob. (512 × 512)**	$p_{\mathrm{sky},512}$	CNN sky probability at input size 512 × 512, resampled to 1024 × 1024.
**Sky prob. (1024 × 1024)**	$p_{\mathrm{sky},1024}$	As above, input size 1024 × 1024.
**Sky prob. (2048 × 2048)**	$p_{\mathrm{sky},2048}$	As above, input size 2048 × 2048.
**RGB channels**	R,G,B	Per-channel intensities.
**HSV channels**	H,S,V	V=max(R,G,B), $S=\frac{V-\min(R,G,B)}{V}$, *H* computed piecewise by dominant channel
YCbCr components	$Y,C_{b},C_{r}$	Y=0.299R+0.587G+0.114B, $C_{b}=0.564\left(B-Y\right)$, $C_{r}=0.713\left(R-Y\right)$
**Patch mean**	$\mu_{\Omega }\left(I\right)$	$\mu_{\Omega }=\frac{1}{\left|\Omega \right|}\sum_{y\in \Omega }I\left(y\right)$ in a 7×7 window.
**Patch std. dev.**	$\sigma_{\Omega }\left(I\right)$	$\sigma_{\Omega }=\sqrt{\frac{1}{\left|\Omega \right|}\sum_{y\in \Omega }{\left(I\left(y\right)-\mu_{\Omega }\right)}^{2}}$.
Dark channel [22]	*D*	$D\left(x\right)=\min_{c\in \{R,G,B\}}I_{c}\left(x\right)$.
Scene depth [22]	$D_{\mathrm{haze}}$	$D_{\mathrm{haze}}=c_{0}+c_{1}V+c_{2}S$, with $\left(c_{0},c_{1},c_{2}\right)=\left(0.1218,\;0.9597,\;-0.7802\right)$.
Hue disparity [22]	ΔH	$\Delta H=|H-H_{\mathrm{si}}|$, where $H_{\mathrm{si}}$ is the hue of the semi-inverse RGB image.
Color saturation [22]	*S*	$S\left(x\right)=1-\frac{\min_{c\in \{R,G,B\}}I^{c}\left(x\right)}{\max_{c\in \{R,G,B\}}I^{c}\left(x\right)}$.
**Contrast energy** [22]	CE	For c∈{gray,yb,rg}, $CE^{c}\left(x\right)=\frac{\alpha \;Z^{c}\left(x\right)}{Z^{c}\left(x\right)+\alpha k}-\tau_{c}$, with $Z^{c}\left(x\right)=\sqrt{{\left(I^{c}\;⊗\;g_{h}\right)}^{2}+{\left(I^{c}\;⊗\;g_{v}\right)}^{2}}$; here ⊗ denotes convolution, $g_{h}$ and $g_{v}$ are the horizontal/vertical second-order derivative-of-Gaussian filters, $\alpha =\max(Z^{c})$, k=0.1, $\tau_{c}=\left\{0.2353,0.2287,0.0528\right\}$, and channels are $I^{gray}=0.299R+0.587G+0.114B$, $I^{yb}=0.5\left(R+G\right)-B$, $I^{rg}=R-G$.
Color gradient [22]	∥∇I∥	$∥\nabla I∥\left(x\right)=\sum_{c\in \{R,G,B\}}\sqrt{{\left(\partial_{x}I_{c}\right)}^{2}+{\left(\partial_{y}I_{c}\right)}^{2}}$ (Sobel operator).
**Uniformity** [23]	U	$U=\sum_{b=1}^{B}P{\left(b\right)}^{2}$, with P(b) histogram probability in bin *b* (B=10).
**Entropy** [23]	$H_{e}$	$H_{e}=-\sum_{b=1}^{B}P\left(b\right)\log P\left(b\right)$.
**Texture** [23]	T	$T=\frac{1}{L-1}\sum_{i=1}^{L}\left|p_{i}-p_{j}\right|$, $p_{j}$ central pixel in patch of size *L*.
Smoothness [23]	$S_{m}$	$S_{m}=1-\frac{1}{1+s^{2}}$, *s* is the standard deviation of the local patch.
Third moment [23]	*t*	$t=\frac{1}{L}\sum_{i=1}^{L}{\left(p_{i}-m\right)}^{3}$, where *m* is the patch mean.
Gradient magnitude [23]	∥∇Y∥	∥∇Y∥=|p(x−1,y)−p(x+1,y)|+|p(x,y−1)−p(x,y+1)|, where p(x,y) is the grayscale intensity at image coordinate (x,y).
**Fisher Discriminant (RGB)** [23]	${\mathrm{FD}}_{\mathrm{RGB}}$	${\mathrm{FD}}_{\mathrm{RGB}}=-3.77R-1.25G+12.40B-4.62$.
**Fisher Discriminant (HSV)** [23]	${\mathrm{FD}}_{\mathrm{HSV}}$	${\mathrm{FD}}_{\mathrm{HSV}}=3.35H+2.55S+8.58V-7.51$.
**Excess blue index** [57]	ExB	ExB=1.4B−G.
Distance to center	*r*	$r\left(x\right)=\frac{\sqrt{{\left(x-x_{0}\right)}^{2}+{\left(y-y_{0}\right)}^{2}}}{R_{\max}}$, with $\left(x_{0},y_{0}\right)$ the disk center and $R_{\max}$ its radius.

**Table 5:** Performance of baseline U-Net++ models with EfficientNet encoders (without LGBM).

Model	F1 (%)	IoU (%)	Boundary F1 (%, τ=3 px)	Boundary IoU (%)
EfficientNet-b4	97.83	95.75	86.54	87.47
EfficientNet-b5	97.76	95.61	86.89	87.78
EfficientNet-b6	97.85	95.78	89.96	88.77
EfficientNet-b7	97.89	95.86	89.38	89.20

**Table 6:** Performance of LGBM-enhanced segmentation models.

Model	F1 (%)	IoU (%)	Boundary F1 (%, τ=3 px)	Boundary IoU (%)
LGBM + EfficientNet-b4	98.24	96.54	91.61	90.77
LGBM + EfficientNet-b5	98.22	96.50	92.03	91.16
LGBM + EfficientNet-b6	98.27	96.60	92.21	91.43
LGBM + EfficientNet-b7	98.29	96.63	92.25	91.03

**Table 7:** IoU comparison of our models evaluated on fisheye projections of the CVRG-Pano dataset.

Model	Post-Processing	IoU (%)
EfficientNet-b4 + U-Net++	None	95.86
EfficientNet-b5 + U-Net++	None	95.48
EfficientNet-b6 + U-Net++	None	95.39
EfficientNet-b7 + U-Net++	None	95.80
EfficientNet-b4 + U-Net++	LightGBM	95.74
EfficientNet-b5 + U-Net++	LightGBM	95.44
EfficientNet-b6 + U-Net++	LightGBM	95.05
EfficientNet-b7 + U-Net++	LightGBM	95.92

**Table 8:** Average per-image runtime (mean ± std, in seconds) for CNN prediction across three input resolutions, handcrafted feature extraction, and LightGBM inference across EfficientNet-b4–b7 backbones, computed over 42 images of size 1024 × 1024.

	CPU (i5-1335U, 1.3 GHz)			GPU (H100 + EPYC 9124)		
Model	CNN (Multi-Scale)	Features	LGBM	CNN (Multi-Scale)	Features	LGBM
EfficientNet-b4	15.10 ± 3.35	1.10 ± 0.43	0.64 ± 0.26	0.33 ± 0.04	0.82 ± 0.08	1.35 ± 0.13
EfficientNet-b5	18.92 ± 3.99	1.24 ± 0.52	0.77 ± 0.28	0.36 ± 0.05	0.81 ± 0.07	1.59 ± 0.13
EfficientNet-b6	24.35 ± 5.19	1.21 ± 0.49	0.65 ± 0.26	0.42 ± 0.04	0.82 ± 0.08	1.29 ± 0.10
EfficientNet-b7	29.13 ± 6.80	1.12 ± 0.47	0.49 ± 0.25	0.50 ± 0.04	0.79 ± 0.07	1.09 ± 0.08

## Data Availability

The data presented in this study are openly available in Fisheye sky segmentation at https://gitlab.laas.fr/nbouillon/fisheye-sky-segmentation (accessed on 1 December 2025).
